# Supplementary material for: Asynchronous magnetic resonance elastography: Shear wave speed reconstruction using noise correlation of incoherent waves
Source: Magn Reson Med. 2022 Oct 27;89(3):990–1001. doi: 10.1002/mrm.29502 (PMC9792433; doi:10.1002/mrm.29502)
Supplement: Supplementary file 1 — DATA S1 MATLAB code used to generate the numerical simulations of Figure 1 [file MRM-89-990-s002.zip › k-Wave/helpfiles/example_tvsp_homogeneous_medium_dipole.html]

Dipole Point Source In A Homogeneous Propagation Medium Example (k-Wave)


# Dipole Point Source In A Homogeneous Propagation Medium Example

This example provides a simple demonstration of using k-Wave for the simulation and detection of a time varying pressure dipole source within a two-dimensional homogeneous propagation medium. It builds on the Monopole Point Source In A Homogeneous Propagation Medium Example.

- Open the file in the MATLAB Editor
- Run the file in MATLAB

## Contents

- Defining the time varying velocity source
- Running the simulation

## Defining the time varying velocity source

A time varying velocity source is defined analogous to the time varying pressure source encountered in the previous example. A binary matrix (i.e., a matrix of 1's and 0's with the same dimensions as the computational grid) is assigned to `source.u_mask`, where the 1's represent the grid points that form part of the source. The time varying input signal is then assigned to `source.ux` and `source.uy`. These can be defined independently, and may be a single time series (in which case the same time series is applied to all source points), or a matrix of time series following the source points using MATLAB's column-wise linear matrix index ordering.

In this example, a dipole is created by assigning a sinusoidal velocity input to a single source point. The input is filtered using `filterTimeSeries` to remove any high-frequency components not supported by the grid.

```
% define a single source point
source.u_mask = zeros(Nx, Ny);
source.u_mask(end - Nx/4, Ny/2) = 1;

% define a time varying sinusoidal velocity source in the x-direction
source_freq = 0.25e6;       % [Hz]
source_mag = 2 / (medium.sound_speed * medium.density);
source.ux = -source_mag * sin(2 * pi * source_freq * kgrid.t_array);

% filter the source to remove high frequencies not supported by the grid
source.ux = filterTimeSeries(kgrid, medium, source.ux);
```

Note, an acoustic dipole can also be created using a pressure source comprising of two adjacent grid points with their inputs out-of-phase. Higher order source patterns can similarly be created using combinations of pressure or velocity sources.

## Running the simulation

A plot of the input time series driving the source along with the acoustic pressure recorded at the sensor point is given below. The magnitude of the velocity input is scaled by the impedance of the medium, so the magnitude of the pressure recorded at the sensor is the same as in the previous example. The final pressure field within the computational domain is also returned by setting `sensor.record` to `{'p', 'p_final'}`.
